# Brachytherapy-based multimodality cancer treatment: a concept and design perspective on devices to address the challenges of localized radiation therapy

**DOI:** 10.3389/fonc.2026.1812448

**Published:** 2026-08-05

**Authors:** Joshua J. Herskovic, Arnold M. Herskovic

**Affiliations:** 1 Department of Surgery, Baptist Health South Florida, Boynton Beach, FL, United States; 2 Department of Radiation Oncology (Emeritus), Rush University Medical Center, Chicago, IL, United States

**Keywords:** brachytherapy, esophageal stent, glioblastoma, interstitial hyperthermia, intracavitary radiation therapy, local drug delivery, medical device design, radiation dose

## Abstract

**Purpose:**

To present a family of novel brachytherapy-based device concepts that address the geometric, dosimetric, and integrative limitations of current localized radiation therapy, aiming to improve local tumor control while reducing collateral exposure. This is a concept and design paper, not a clinical or dosimetric validation study.

**Methods:**

Multiple device designs were developed over more than a decade, focused on two prototypical clinical settings: intraluminal applicators for the esophagus and intracavitary applicators for the post-resection glioblastoma cavity. Designs include hollow rectangular ribbon (HRR) catheters that encase fluid, gel, or wire isotope sources; modified stents with anti-slippage geometry, dynamic flares, and reservoirs; and a multifunctional cavity device combining brachytherapy with continuous hyperthermia and pressure-modulated diffusional drug, immunotherapy, and nanoparticle delivery. Bench prototypes were constructed and evaluated.

**Results:**

Bench observations demonstrated manipulation of a thermal gradient by modulating the warm source, and an approximately 15-fold increase in dislodgement force for a representative anti-slippage geometry. The designs are further intended to flatten the near-surface dose gradient by expanding the effective source distribution and to integrate drainage, infusion, and pressure modulation in a single device; these dosimetric and integrative benefits are design expectations not yet experimentally tested.

**Conclusion:**

These concepts may improve local control where recurrence concentrates near the resection or stented surface, as in post-resection glioblastoma and obstructive luminal malignancies. The integration of continuous hyperthermia, local diffusion of chemotherapeutic, immunotherapeutic, and protective agents, and pressure-modulated nanoparticle delivery is particularly attractive. The concepts are intended to stimulate further bench, computational, animal, and clinical investigation.

## Introduction

The evolution of tagged radioisotopes, immunotherapy, modern chemotherapy, and increased patient longevity has heightened the importance of effective local therapies, principally surgery and radiotherapy. Brachytherapy is a well-established form of local therapy whose central appeal is geometric: very high local doses can be delivered with steep fall-off into surrounding normal tissue. Despite this advantage, several limitations persist: non-uniform near-surface dose with potentially excessive Dmax, sensitivity of dose distribution to applicator geometry, and the difficulty of integrating brachytherapy with other modalities such as hyperthermia and drug delivery. We believe these limitations represent design opportunities.

Two clinical settings illustrate both the unmet need and the design challenge. The first is the obstructive distal esophageal cancer, where palliative or definitive stent placement is common and where local recurrence (often near or across the gastroesophageal junction) remains a dominant cause of failure (1–4). The second is the post-resection glioblastoma cavity, where most recurrences occur within 0.5 to 1.0 cm of the resection rim, and where outcomes from external beam radiotherapy (including stereotactic, intensity-modulated, and neutron techniques), altered fractionation, and chemotherapy have been disappointing (5, 6). The recently approved Gamma Tile Cs-131 collagen-tile system (surgically targeted radiation therapy, STaRT) demonstrates the clinical attractiveness of intracavitary brachytherapy in this setting (7–9), and motivates further device innovation.

This paper introduces a family of device concepts intended to address these challenges. We focus on three design elements: (i) hollow rectangular ribbon (HRR) catheters that expand the effective source distribution and thereby flatten the near-surface dose gradient; (ii) anti-slippage and conformal stent geometries with multi-purpose apertures and reservoirs; and (iii) a multifunctional post-resection cavity device that combines brachytherapy with continuous hyperthermia and pressure-modulated diffusion of chemotherapeutic, immunotherapeutic, sensitizing, protective, and nanoparticle agents. The work is presented as a concept and design perspective; bench, computational, animal, and clinical validation studies are required and are identified as future directions.

## Methods: device designs and bench setup

### Brachytherapy modalities and source considerations

Brachytherapy is most often delivered using high dose rate (HDR), low dose rate (LDR), or very low dose rate (vLDR, often permanent) sources. HDR offers superior radiation safety and dose-delivery assurance but is intermittent. Conventional sealed sources include filtration that attenuates undesirable lower-energy emissions. The most commonly used isotopes are I-125 and Cs-131 for permanent implants and Ir-192 for HDR. Other isotopes, for example Yb-169 (mean photon energy ~ 93 keV, half-life ~ 32 days, remain of interest for specific applications.

HDR with Ir-192 requires an external after loader and a shielded room because of the high source activity. Liquid isotopes simplify conformal source distribution but introduce risks of leakage, personnel exposure, and contamination. This can be mitigated by using many dwell points with HDR. An alternative is the use of Ir-192 wires or sealed seeds in catheters that allow continuous delivery and can be combined with concurrent modalities such as hyperthermia. Surface “painting” of a stent with a sealant containing isotope is a further alternative but requires pre-planning and is not readily modified in clinical practice.

### Hollow rectangular ribbon catheters and effective source distribution

**Figure 1:**
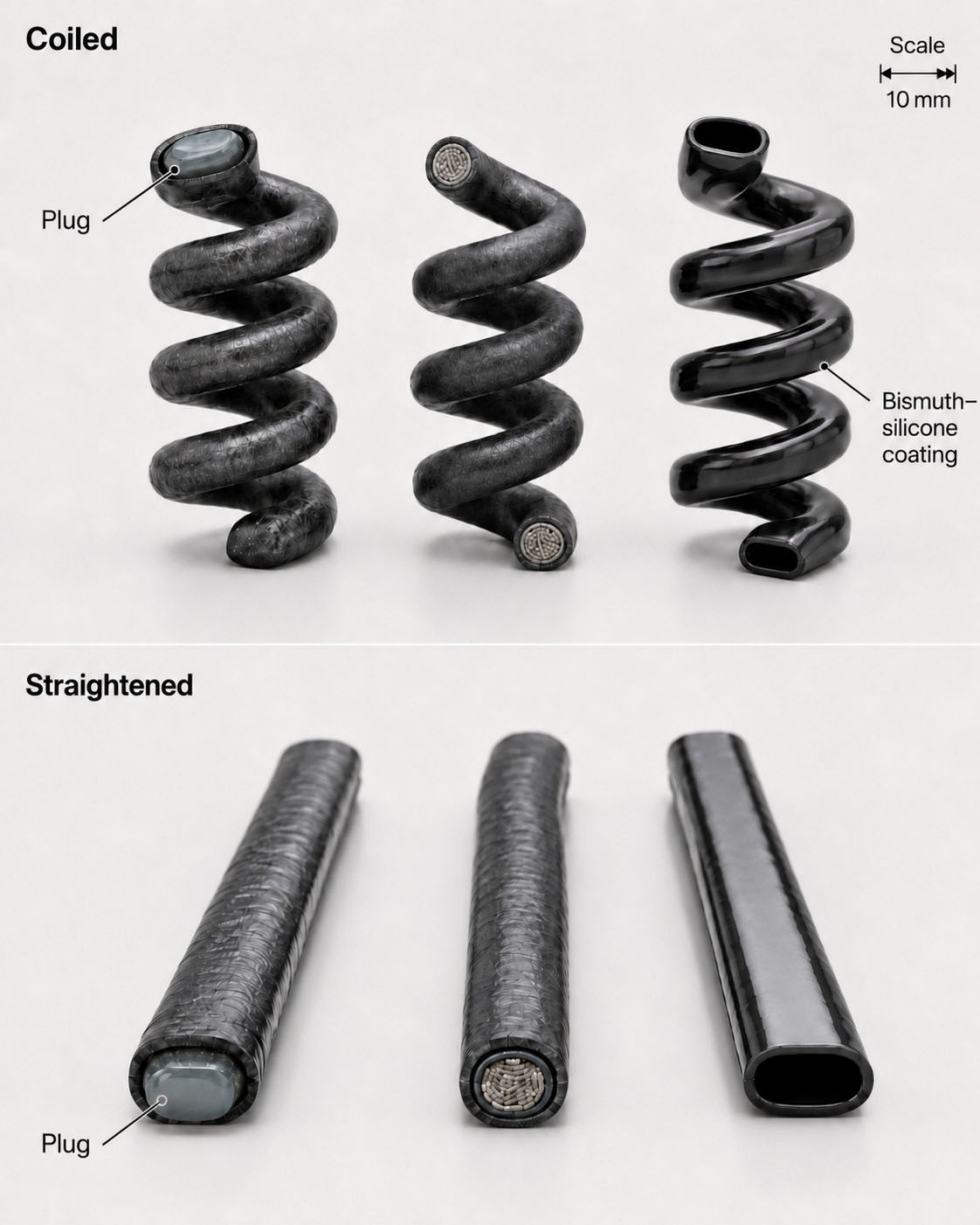
Hollow rectangular ribbon (HRR) catheter concepts, shown coiled (upper panel) and straightened (lower panel) with the proximal cross-section visible in each. The three variants illustrate the progression from a conventional round profile to the proposed rectangular geometry. The center specimen is a nitinol coil with a circular cross-section, representative of the round lumen of most conventional catheters. The specimen on the right is nitinol with an approximately rectangular cross-section and an open lumen; the specimen on the left is of similar rectangular geometry but is fitted with a plug (grey, at the distal end). Nitinol was selected for its biocompatibility and fatigue resistance. The rectangular lumen is designed to hold a fluid (liquid, gel, or similar medium) in which the radioactive source can be distributed, spreading the activity along the ribbon rather than concentrating it at a point; this expanded source distribution is expected to reduce the near-surface maximum dose (Dmax) and thereby improve normal-tissue tolerance. All specimens are coated with a silicone–high-atomic-number composite that attenuates the low-energy, non-therapeutic component of the emission spectrum; a bismuth–silicone mixture is shown here, although other materials may prove more practical. Scale: the coil outer diameter is approximately 15 mm.

To address the high near-surface Dmax associated with point-like seed sources, we designed hollow rectangular ribbon (HRR) catheters fabricated from nitinol with a bismuth-silicone sealant (**Figure 1**). HRR catheters can encase fluids, gels, or malleable wires containing isotopes with appropriate filtration. Distributing the activity along the ribbon expands the effective source distribution, the spatial extent over which the activity is arranged near the applicator surface, and thereby flattens the radial dose gradient relative to a point or near-point source delivering the same prescribed dose at depth.

This is dosimetrically distinct from interstitial implant geometry. The relevant quantity for an intracavitary or intraluminal applicator is the radial radiation dose gradient measured outward from the applicator surface, which is governed primarily by inverse-square fall-off and by the activity nearest the surface. Standard interstitial coverage indices (D90, V100) and conformity indices, developed for anatomically defined target volumes (prostate, head and neck, breast interstitial), are not the appropriate quality metrics for this device class. The relevant comparators are vaginal cylinders, intrabronchial applicators, balloon brachytherapy devices (MammoSite, GliaSite), and the GammaTile Cs-131 system. For these applicators, near-surface Dmax is dominated by activity in the immediately adjacent volume; contributions from more distant sources are reduced by distance and by attenuation through the applicator structure. Increasing the number of catheters and HDR dwell positions, or distributing activity along an HRR catheter, expands the effective source distribution and reduces Dmax at the applicator surface for the same prescribed dose at the typical 0.5 to 1.0 cm depth. Rivard reported an approximately 25% Dmax reduction for the spheroidal GliaSite model when low-energy I-125 and Cs-131 sources were used in an expanded distribution (M.J. Rivard, personal communication). It should be emphasized that, for the HRR and multichannel concepts introduced here, the expected reduction in near-surface Dmax and flattening of the radial dose gradient are theoretical design expectations based on established brachytherapy physics; no dosimetric simulation or experimental dose measurement is presented in this concept paper.

### Stent designs: anti-slippage, dynamic flares, and reservoirs

**Figure 2:**
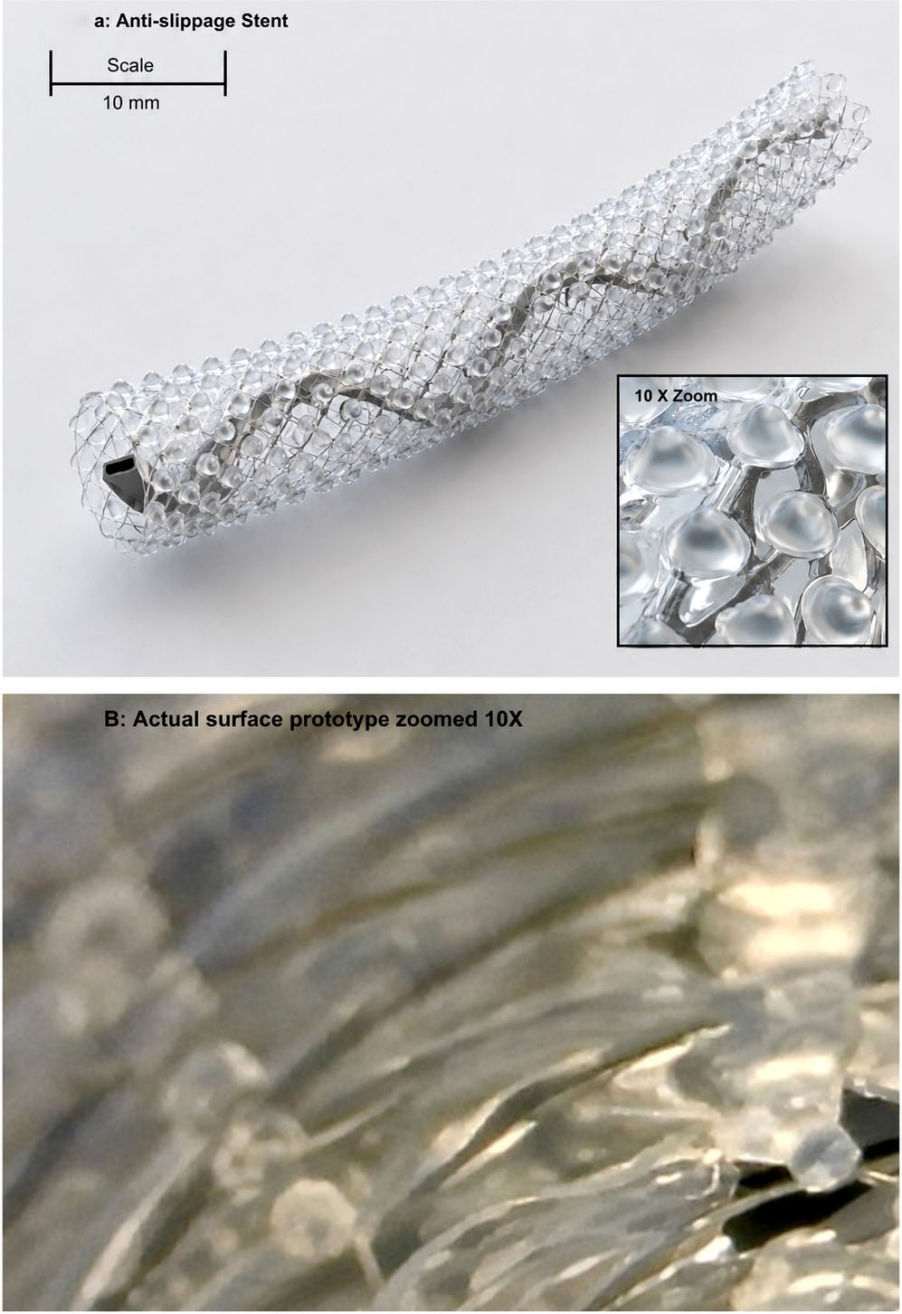
Anti-slippage stent prototype. On bench testing, the axial dislodgement force increased from approximately 10 N for a conventional-geometry baseline stent to approximately 150 N for the anti-slippage geometry (an approximately 15-fold increase), measured with a force transducer placed in line with the axial pull (five repeated pulls per specimen; one specimen of each design; ten pulls total; Kellogg’s Research Laboratory). These are preliminary single-specimen measurements; a formal multi-specimen study is required for confirmation. CAD-rendered illustration showing the anti-slippage geometry (blunted cones between the stent arms and the dynamic flares), with component labels. **(A)** CAD rendering of stent with Anti-slip coating with inset 10x rendering of close up of anti-slip coating, **(B)**, closeup of actual stent surface used in the experiment.

Esophageal stent slippage remains a clinically significant problem, particularly at the gastroesophageal junction where the lumen and tumor geometry change rapidly. We developed prototype stents incorporating dynamic flares at both ends, hybrid surface geometry with multi-purpose apertures and reservoirs, and a means for early slippage detection. A representative prototype with silicone-blunted cones between stent arms (**Figure 2**) showed an approximately 15-fold increase in the force required for dislodgement on bench testing. Practical considerations suggest that vLDR or permanent radiation sources may have greater clinical acceptance than HDR for these stents.

### Post-resection cavity device: integrated brachytherapy, hyperthermia, and diffusion

**Figure 3:**
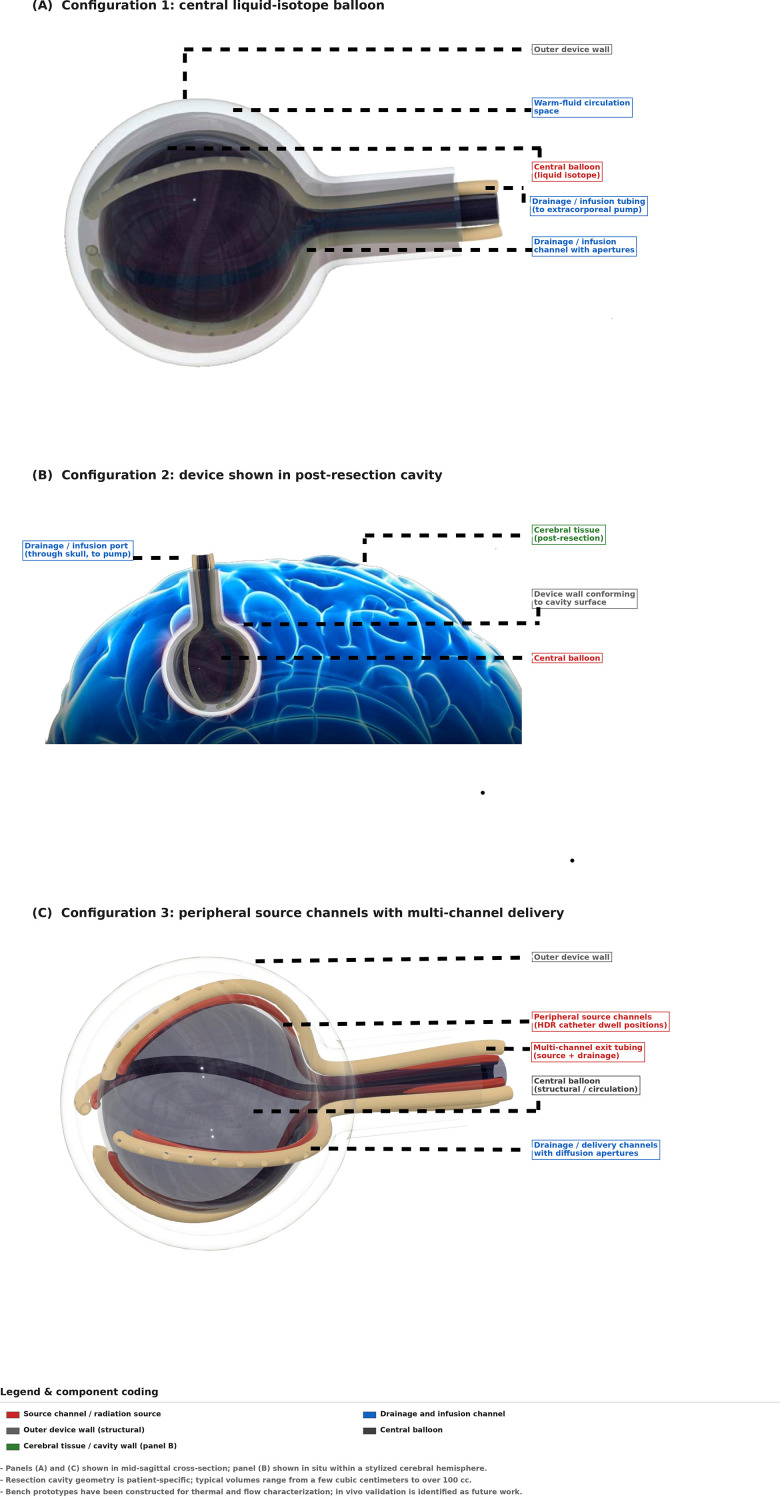
Schematics of three iterations of the multifunctional post-resection cavity device. **(A)** Liquid isotope in a central balloon, surrounded by a circulating warm-fluid space; structural integrity is provided by the drainage tubes. **(B)** Central warm-fluid lumen with peripheral drainage catheters. **(C)** Radiation sources placed in a catheter attached to the drainage tube on the side opposite the balloon wall, providing additional separation between the source and the cavity wall.

For the post-resection cavity (the GBM example), we designed a multifunctional applicator that combines brachytherapy with continuous hyperthermia and pressure-modulated drainage and infusion (**Figure 3**). Several configurations were prototyped: a central balloon containing liquid isotope surrounded by a circulating warm-fluid space (**Figure 3A**); a central warm-fluid lumen with peripheral drainage catheters (**Figure 3B**); and a configuration in which radiation sources are placed in a catheter attached to the drainage tube on the side opposite the balloon wall (**Figure 3C**). The device structure is held together by drainage tubes, which also serve as the conduits for fluid circulation, infusion, and suction.

An intrinsic drain creates both structure and spacing, e.g. ½ cm, between the radiation sources and the external wall of the device. The spacing flattens the near-source dose gradient and removes any intervening fluid that might otherwise create a “cold” spot. In current designs, drainage channels are positioned adjacent to or integrated into the wall of the central balloon, with the HDR/LDR catheters positioned on the side opposite the balloon to ensure adequate spacing.

The drainage and infusion ports communicate with an extracorporeal pump. This allows the operator to (i) infuse hypo- or hyper-osmotic solutions, steroids, or therapeutic agents into the cavity, or (ii) apply gentle suction to remove edema fluid, blood, or excess delivered material. By alternating between positive (infusion) and negative (suction) pressures, both osmotic and hydrostatic gradients across the cavity wall can be modulated bidirectionally. This is clinically relevant because the post-resection cavity changes shape and volume over the first several days post-operatively as edema and surgical bleeding evolve, consistent with the frequent daily MRI observations reported by Mehta et al. (10).

### Bench thermal measurements

Preliminary thermal measurements were performed to determine whether the radial thermal gradient produced by the device could be controlled by modulating the warm source. These were proof-of-principle bench measurements conducted with readily available equipment and were not intended as a calibrated thermometric study.

The device was simulated using metallized polyester (Mylar) balloon material positioned within a cavity carved into a water-saturated natural sponge, which served as the facsimile brain tissue. The heat source was a commercially available immersion (coffee-cup) water heater placed inside the balloon; it heated the static water contained within the balloon and was controlled manually by connecting and disconnecting power, with warm (heating) and cool (maintenance) phases alternated at approximately 15-second intervals. The water within the balloon was not circulated in this bench setup.

To simulate the convective heat sink of cerebral perfusion, the sponge-and-cavity assembly was immersed in a circulating water bath maintained at approximately 38 °C, corresponding to normal brain temperature (commonly cited as approximately 37 °C, with some reports slightly higher). The physiological basis for this heat sink is that the brain receives a substantial fraction of cardiac output (on the order of 15–25%, approximately 1.5 L/min for a nominal cardiac output of 6 L/min), distributed across a supratentorial brain volume of approximately 1000 cc (approximately 500 cc per hemisphere); infratentorial structures were excluded because glioblastomas rarely arise in, or are resected from, those regions.

Heat conducted from the internal source water across the Mylar wall into the surrounding sponge, establishing a radial temperature gradient. Temperatures were recorded with commercially available digital thermometers at multiple positions: within the balloon adjacent to the heating element (the source), and at rim and distal-rim positions within the surrounding sponge (**Table 1**). The intended target for the tissue immediately outside the balloon was 43 °C. Because the heater lacked closed-loop feedback control, the source water exceeded this target; the highest readings (approximately 46–47 °C) were recorded by the sensor within the balloon adjacent to the heating element, while positions further into the surrounding sponge recorded progressively lower temperatures approaching the approximately 38 °C background, consistent with the expected radial gradient. The ability to manipulate this gradient by adjusting or holding the thermal source was demonstrated.

**Table 1:** Representative bench thermal measurements during alternating warm (heating) and cool (maintenance) phases, in °C.

Phase (Cycle)	In-balloon source (°C)	Proximal rim (°C)	Mid rim (°C)	Distal rim (°C)
Heat (1)	46.3	46.7	39.4	38.1
Cool (1)	38.1	37.5	35.5	—
Heat (2)	45.6	41.7	42.2	38.2
Cool (2)	38.1	38.8	38.7	38.3
Heat (3)	46.7	38.8	38.0	37.1

The heat source was an unregulated immersion heater within the simulated balloon; the intended target for the surrounding tissue was 43 °C. The in-balloon source readings (approximately 46–47 °C during heating) were recorded adjacent to the heating element and reflect the source water temperature itself, which exceeded the 43 °C tissue target owing to the absence of closed-loop feedback control. Rim and distal-rim positions, located within the surrounding water-saturated sponge, show the expected decline toward the approximately 38 °C circulating background that simulated cerebral perfusion. Temperatures were measured with commercially available digital thermometers. These are preliminary proof-of-principle measurements; a controlled study with closed-loop feedback thermometry is required for confirmation.

These observations require confirmation in a controlled, instrumented apparatus. In a clinical device the internal fluid would be actively circulated and thermostatically regulated; thus, homogenizing the intraluminal temperature and compensating for perfusion heat loss. The incorporation of miniature thermometers in a closed feedback loop would hold the source at the intended target and eliminate source-proximal overshoot. These refinements are identified as required next steps.

## Results: bench observations

The bench observations support three principal design claims. First, the temperature near the thermal source can be reduced more rapidly than at the mid and distal rim, demonstrating effective control of the near-surface thermal gradient. Alternating between uniformly warm fluid (43 °C) and slightly cooler fluid (38 °C) reduces the peak central-to-rim gradient. Second, continuous warm-fluid circulation counteracts convective thermal loss; a feedback temperature loop is therefore a reasonable next refinement. Third, on bench testing, the axial force required to dislodge the stent increased from approximately 10 N for a conventional-geometry (baseline) stent to approximately 150 N for the anti-slippage geometry — an approximately 15-fold increase. Force was measured with a transducer placed in line with the axial pull; a single specimen of each design was tested with five repeated pulls (ten pulls in total), performed at Kellogg’s Research Laboratory. Because a single specimen of each design was tested, these values characterize the difference between these two prototypes rather than a population; a formal multi-specimen study is identified as a required next step.

**Figure 4:**
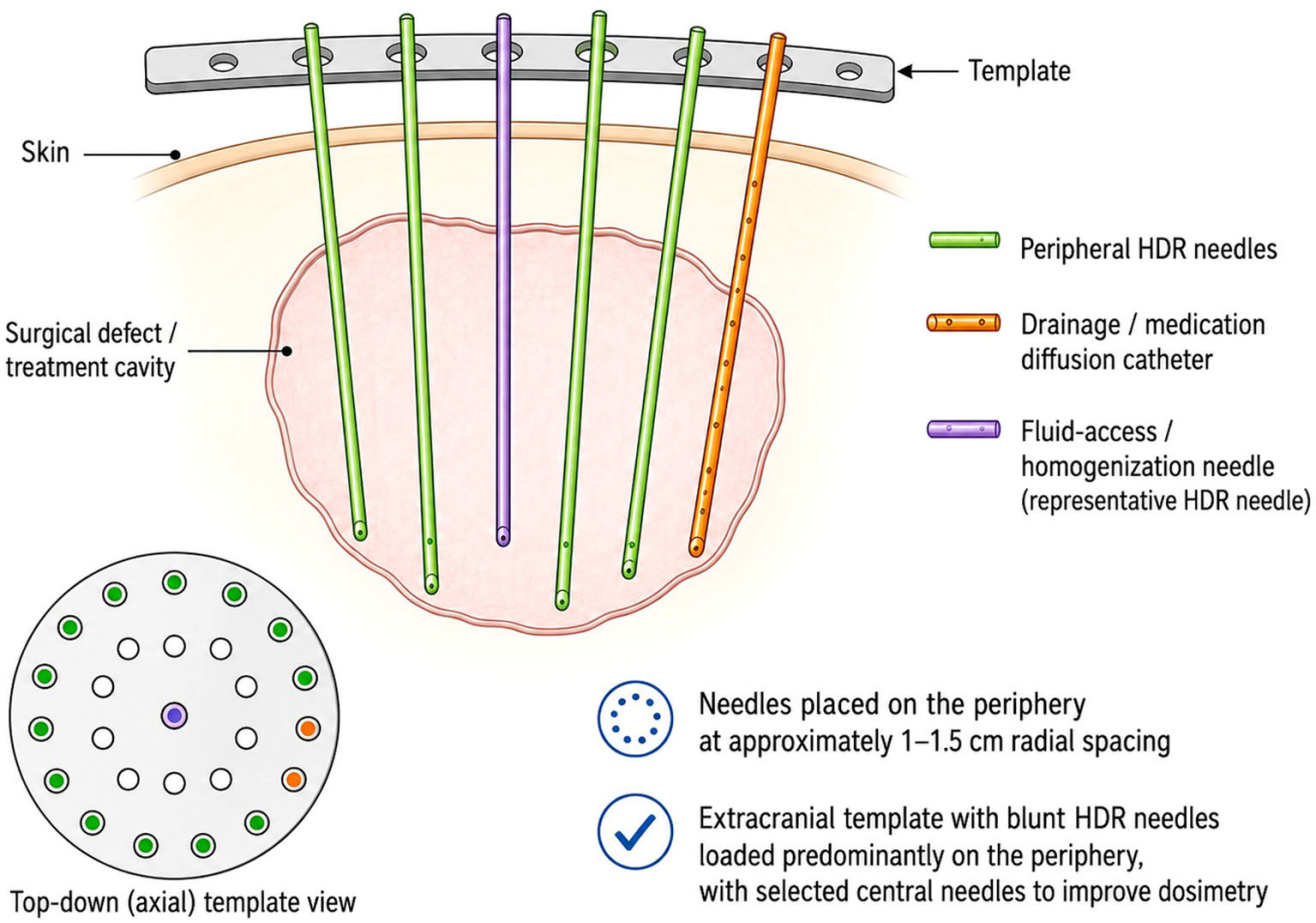
Side/elevation view of an HDR brachytherapy template concept: Blunt HDR after-loading needles are positioned through the template and arranged predominantly around the periphery of the treatment cavity, with optional central loading to improve dosimetry. Peripheral source dwell positions may be selected to deliver a conformal, homogeneous dose to the tumor bed while improving dose falloff away from adjacent tissue. A spacer, drain, or fluid-access catheter may be placed between the device wall and source needles to further improve dose gradient, drainage, and medication/fluid diffusion.

Conformal device prototypes were developed and evaluated for the integration of source enhancements (HRR catheters, multiple dwell positions, expanded effective source distribution) intended to flatten the radial radiation dose gradient in the post-resection GBM example. Drainage of intervening fluid, which would otherwise create a “cold” spot, was demonstrated using the multifunctional drainage channels. A template-based arrangement of peripheral HDR after-loading needles, with optional central loading to improve dosimetry, illustrates this approach (**Figure 4**).

## Discussion

### Dosimetric considerations for intracavitary and intraluminal applicators

The devices presented in this paper are intracavitary or intraluminal applicators. As discussed in the Methods, the appropriate dosimetric metrics for this class of applicator are the radial radiation dose gradient and the near-surface Dmax, not the interstitial coverage indices (D90, V100) and conformity indices that were developed for anatomically defined interstitial target volumes. Dmax at the applicator surface is dominated by activity nearest the surface; sources further away contribute less by distance and by attenuation through the applicator structure. Adding uniform spacing between the sources effectively flattens the gradient. For HDR applications, increasing the number of catheters as well as centralized sources and dwell positions can act as a replacement for a fluid isotope and substantially improve the gradients. Reducing Dmax for a given prescribed dose at depth can reasonably be expected to reduce local adverse effects, which in turn may permit dose escalation at the typical 0.5 to 1.0 cm target depth and so address the dominant local-recurrence pattern in glioblastoma (11) and in other tumor types where recurrence concentrates near the resection or stented surface. Resection cavity geometry is highly patient-specific (volume from a few cubic centimeters to over 100 cc, irregular shape, evolving over the first several post-operative days as edema resolves) (10), so a single representative simulation would be of limited value; patient-specific dosimetric modeling is identified as a required next step. Contemporary multi-channel breast brachytherapy applicators -- Contura, SAVI, and the MammoSite multi-channel catheter -- demonstrate this principle in the partial-breast irradiation setting: multiple peripheral source channels with independent dwell-time optimization shape the dose distribution and reduce the near-applicator Dmax relative to a single central source. The HRR catheter and multi-channel cavity device concepts proposed here apply the same principle to anatomic settings, the post-resection glioblastoma cavity and the obstructed esophageal lumen, where existing breast applicators are not anatomically suitable. These dosimetric benefits remain design expectations at this stage and require confirmation by patient-specific dosimetric modeling and experimental dose measurement.

### Hyperthermia

Clinical hyperthermia, particularly in combination with radiation, has consistent supporting evidence in cervical cancer, breast cancer, and other settings (12–14). Practical limitations have included intermittent delivery, spatial heterogeneity of heating, and difficulty with thermometry. The device presented here is designed to deliver continuous, instrumented hyperthermia in the post-resection cavity (or in an analogous luminal applicator), with thermometry from miniature transducers placed through the device’s auxiliary channels and with continuous warm-fluid circulation to counteract convective loss. Thermal conduction depends on contact area, temperature differential, and the specific heat of the involved materials; all but volume can be modulated by the device. Increasing local barometric pressure can theoretically enhance conductivity, but the clinically tolerable range is uncertain.

### Multimodality therapy and tumor microenvironment barriers

A device that provides physical access to the resection cavity or to the luminal surface allows local diffusion of chemotherapeutic, immunotherapeutic, gene-based sensitizing agents, and protective agents directly into the target volume, with reduced collateral tissue exposure. The principal physiological barrier to such delivery is the well-described elevated tumor interstitial fluid pressure (IFP), driven by abnormal and hyperpermeable tumor vasculature, dense and disorganized extracellular matrix (ECM), and impaired lymphatic drainage (15, 16). Elevated IFP opposes convective transport of drugs and macromolecules from the vasculature into the tumor interstitium and is now recognized as a dominant barrier in many solid tumors (15). Strategies to overcome this barrier include vascular normalization, modulation of the ECM, ultrasound-based pressure modulation, and direct local administration that bypasses the vascular route (15–17).

Nanoparticle delivery is influenced by particle size, surface charge (zeta potential), shape, and the dimensions of the local extracellular space (17, 18, 20, 21). The enhanced permeability and retention (EPR) effect, originally proposed as a passive-targeting mechanism, provides only modest selectivity in many human solid tumors and is heterogeneous across patients and tumor types (18, 19). Earlier nanoparticle-mediated hyperthermia efforts achieved limited intracellular accumulation (P. Stauffer, personal communication). The multifunctional cavity device addresses these barriers with a fundamentally different strategy: rather than relying on systemic delivery and the EPR effect, agents are introduced directly into the cavity through multiple delivery apertures, and local pressure is modulated bidirectionally to drive convective transport into the surrounding tissue against the residual interstitial pressure gradient. The approach is compatible with simultaneous brachytherapy and continuous hyperthermia, both of which can independently increase local tumor perfusion and permeability (14, 18).

### Future directions

The concepts presented here require validation through several distinct lines of work, identified explicitly: (i) device-specific Monte Carlo dosimetric modeling once a prototype is finalized; (ii) bench measurements of thermal gradient, drug penetration, and pressure-modulated transport in tissue-simulating phantoms and ex vivo tissue; (iii) animal studies in appropriate models of glioblastoma and obstructive luminal cancer; (iv) refinement of nanoparticle formulations matched to the device’s diffusion and pressure-modulation characteristics; and (v) ultimately, first-in-human studies in carefully selected indications. The integration of continuous hyperthermia, local diffusion of agents, and bi-directional pressure modulation in a single device is a deliberately ambitious scope and will require collaboration across radiation oncology, neurosurgery, surgical oncology, medical physics, biomedical engineering, and pharmaceutical sciences.

### Limitations

This paper is a concept and design perspective. It does not include patient-specific dosimetric simulations, *in vivo* pharmacokinetic data, or clinical outcomes. Resection cavity geometry varies widely between patients and evolves over time, so a single dosimetric simulation would not generalize. Detailed nanoparticle transport simulations are highly geometry-dependent and beyond the scope of this work. Bench thermal data presented here are from prototype devices in tissue-simulating phantoms and have not yet been replicated *in vivo*. Several of the integrative concepts presented are novel and therefore lack a direct clinical track record by definition; each underlying mechanism is, however, supported by the cited literature.

## Conclusion

We present a family of brachytherapy-based device concepts intended to address the geometric, dosimetric, and integrative limitations of current localized radiation therapy. The core design elements (hollow rectangular ribbon catheters that expand the effective source distribution and flatten the near-surface dose gradient; anti-slippage and conformal stent geometries; and a multifunctional post-resection cavity device that combines brachytherapy with continuous hyperthermia and bidirectional pressure-modulated diffusion of therapeutic agents) are intended to stimulate further bench, computational, animal, and clinical work. The integration of continuous hyperthermia and local pressure-modulated drug and nanoparticle delivery within a brachytherapy device is, to our knowledge, novel, and may prove clinically useful where local recurrence dominates outcomes.

Brachytherapy presents unique radiation safety challenges for patients, medical staff, and ancillary personnel, and these challenges shape every aspect of device design. Tumor geometry varies widely between patients and disease sites, so no single applicator configuration will be sufficient; the concepts presented here are intended as a flexible framework rather than a single prescriptive design. An optimistic “wish list” of candidate applications spanning multiple anatomic sites and disease settings is included as **Table 2**, limited only by clinical creativity. This paper is intended to stimulate discussion and collaboration across radiation oncology, surgery, medical physics, biomedical engineering, and pharmaceutical sciences.

**Table 2:** Candidate clinical applications for the proposed brachytherapy-based multimodality device family.

Site/Disease	Comments	HDR	LDR	Perm vLDR	BTX	Hypertx	Drain	Chemo	Immunotx	Notes	Enthusiasm
Brain											
High-grade gliomas	*Multimodality boost*	Y	Y	Y	Y	Y	Y	Y	?		HIGH
Meningiomas, other	*Post-surgery*	Y	Y	Y	Y	N	Y	N	N		INTERMED
Spine/Other											
Kyphoplasty	*Many iterations*	N	N	Y	Y	N	N	N¹	N		HIGH
Long Bones	*Alternative to EBRT (External Beam Radiation)*	N	N	Y	Y	N	N	N	N		INTERMED
Breast											
Breast (general)	*Many options*	Y	Y	Y	Y	Y	Y	Y	HORM		HIGH
Chest wall	*Needs creativity*	Y	Y	N	Y	Y	N	Y	HORM		HIGH
LDR sources not widely available.											
Head & Neck											
Post-neck dissection	*—*	Y	N	Y	Y	Y	Y	N	N	US interstitial	HIGH
Oral cavity/tongue	*—*	Y	N	Y	Y	N	Y	N	N	US interstitial	HIGH
Thorax											
Bronchogenic	*Convert unresectable to resectable*	Y	N	Y	N	Y	N	Y	N		INTERMED
Bronchus/trachea	*Luminal*	Y	N	Y	Y	Y	N	Y	N	Esp. carinas	INTERMED
Mesothelioma	*—*										
Gastrointestinal											
Esophagus	*Could be permanent*	Y	Y	Y							
Gastric/gastric outlet	*Few alternatives*	Y	N	Y	Y	Y	Y	Y	Y	Needed	HIGH
Rectum	*Often unique*	Y	N	Y	Y	Y	Y	Y	N	Probable	HIGH
Pelvic/Gynecologic											
Cervix	*Alternatives*	Y	N	Y	Y	Y	Y	N	N		HIGH
Peritoneal surface	*Creative planar*							Y	Y	Needs creativity	
Urogenital											
Ureter (rare)	*Luminal*	N	N	Y	Y	N	Y	N	N		INTERMED
Pancreatic											
Pancreatic ducts	*Luminal*	N	N	Y	Y	Y	Y	Y	N		INTER

Anatomic sites and disease settings are grouped by region. For each site, the authors indicate whether each modality is currently applicable (Y), not currently applicable (N), or of uncertain applicability (?). Empty cells denote section headers or sites for which a per-modality assessment was not made. The Enthusiasm column reflects the authors’ subjective clinical priority for further device-focused investigation.

HDR, high dose rate brachytherapy; LDR, low dose rate brachytherapy; Perm vLDR, permanent/very low dose rate brachytherapy; BTX, brachytherapy device or boost (general); Hypertx, hyperthermia; Drain, device-mediated drainage/pressure modulation; Chemo, local chemotherapy delivery; Immunotx, local immunotherapy delivery; HORM, hormonal therapy as an adjunct.

? = applicability uncertain; ¹ = possibly applicable in the future, tentatively classified as no at present; — = section header or category placeholder. HIGH = strong unmet need with reasonable device fit; INTERMED/INTER = promising but more contingent.

## Data Availability

The original contributions presented in the study are included in the article/**Supplementary Material**. Further inquiries can be directed to the corresponding author.
