# Supplementary material for: Brachytherapy-based multimodality cancer treatment: a concept and design perspective on devices to address the challenges of localized radiation therapy
Source: Front Oncol. 2026 Aug 5;16:1812448. doi: 10.3389/fonc.2026.1812448 (PMC13485591; doi:10.3389/fonc.2026.1812448)
Supplement: Supplementary file 1 [file DataSheet1.pdf]

Opportunities to improve patient care	Work in progress subject to continuous improvement									
Site/Disease:	Comments	HDR	LDR	Perm vLDR	BTX	Hypertx	Drain	Chemo	Immunotx	Notes
brain	usually boost									
glioblastoma	multimodality boost	Y	Y	Y	Y	y	Y	Y	pending	
high grade gliomas	multimodality boost	Y	Y	Y	Y	Y	Y	Y	?	
intermed/low grade gliomas	primary or boost	Y	Y	Y	Y	Y	Y	Y	?	
meningiomas other	post surgery	Y	Y	Y	Y	N	Y	N	N	
others										
bone										provocat
kyphoplasty	many iterations	N	N	Y	Y	N	N	N 1	N	
long bones	provocative	N	N	Y	Y	N	N	N	N	
others										
heterotrophic ossification	planar +/-	Y	Y	Y	Y	N	N	N	N	PROB NO
others										
breast	many options									
early resected	permanent implant	Y	Y	Y	Y	Y	Y	Y	HORM	good alte
chest wall	needs cretivity	Y	Y	N	Y	Y	N	Y	HORM	
others										
LDR SOURCES NOT WIDELY AVAILABLE										
Head and Neck	USUALLY INTERSTITIAL									
post neck dissection		Y	N	N	Y	Y	Y	N	N	US INTER
nasopharnyx	CAVITARY	Y	N	Y	Y	N	N	N	N	
oral cavity tongue		Y	N	Y	Y	N	N	Y	N	US INTER
bronchogenic										
convert unrsectable to resectable	eg carina	Y	N	Y	Y	Y	N	Y	N	
bronchius/trachea	luminal	Y	N	N	Y	Y	N	Y	N	ESP CARI
surgical margins	cavitary	Y	N	Y	Y	Y	N	Y	N	CAVIARY
MESOTHELIOMA										
GI tract										
esophagus	COULD BE PERM	Y	Y	Y						
eg junction	FEW ALTERNATIVES	Y	N	Y	Y	Y	Y	Y	Y	NATURA
gastric/gastric outlet	FEW ALTERNATIVES	Y	N	Y	Y	Y	Y	Y	Y	NEEDED
colon	FEW ALTERNATIVES	Y	N	Y	Y	Y	Y	Y	N	NEEDED
rectum	OFTEN UNIQUE	Y	N	Y	Y	Y	Y	Y	N	PROB
anal	UNIQUE	Y	N	Y	Y	Y	Y	Y	N	SPHINCT
gynecological										
cervix	ALTERNATIVES	Y	N	Y	Y	Y	Y	N	N	
vagina	ALTERNATIVES	Y	N	Y	Y	Y	Y	N	N	
peritoneal surface	CREATIVE PLANAR									
		HDR	LDR	VLDR	BTX	HYTX	DRAIN	CHEMO	IMMUNO	
urogenital										
kidney-nephron sparing surgery	RESECTION MARGINS	N	N	Y	Y	Y	Y	N	N	MARGIN
ureter -rare	LUMINAL	N	N	Y	Y	N	Y	N	N	
prostate bed	RESECTION BED	Y	N	Y	Y	Y	Y	N	N	BALLOON
hepatic biliary system										
pancreatic ducts	LUMINAL	N	N	Y	Y	Y	Y	Y	N	
vascular to convert to resectable	LUMINAL	N	N	Y	Y	Y	N	N	N	
SUBSCRIBES 1=POSSIBLY IN THE FUTURE TENTATIVELY NO										
2- MEANT AS ADJUNCT TO EXTERNAL BEAM										
3- THIS WOULD BEVERAL ALTERNATE TREATMENT HAVE BEEN USED BUT										
THIS WOULD BE MORE CONFORMAL AND MINIZE SURGICAL DEFECT										
CONVENTIONAL LDR SOURCES NOT WIDELY AVAILABLE BUT LIQUID RADIO										
ISOTOPES PRESENT OVER A FEW DAYS IS A LDR TREATMENT										
