# Supplementary material for: Brachytherapy-based multimodality cancer treatment: a concept and design perspective on devices to address the challenges of localized radiation therapy
Source: Front Oncol. 2026 Aug 5;16:1812448. doi: 10.3389/fonc.2026.1812448 (PMC13485591; doi:10.3389/fonc.2026.1812448)
Supplement: Supplementary file 2 [file DataSheet2.pdf]

Appendix: Potential Applications for Brachytherapy-Based Multimodality Devices

A clinician's wish list of candidate sites and modalities for further investigation

Opportunities to improve patient care — *Work in progress, subject to continuous improvement*

Site / Disease	Comments	HDR	LDR	Perm vLDR	BTX	Hypertx	Drain	Chemo	Immunotx	Notes	Enthusiasm
**Brain**											
**High-grade gliomas**	*Multimodality boost*	Y	Y	Y	Y	Y	Y	Y	?		HIGH
**Meningiomas, other**	*Post-surgery*	Y	Y	Y	Y	N	Y	N	N		INTERMED
**Spine / Other**											
**Kyphoplasty**	*Many iterations*	N	N	Y	Y	N	N	N[1]	N		HIGH
**Others**	—										
**Breast**											
**Breast (general)**	*Many options*										
**Chest wall**	*Needs creativity*	Y	Y	N	Y	Y	N	Y	HORM		HIGH
LDR sources not widely available.											
**Head & Neck**											
**Post-neck dissection**	—	Y	N	Y	Y	Y	Y	N	N	US interstitial	HIGH
**Oral cavity / tongue**	—	Y	N	Y	Y	N	Y	N	N	US interstitial	HIGH
**Thorax**											
**Bronchogenic**	—										
**Bronchus / trachea**	*Luminal*	Y	N	Y	Y	Y	N	Y	N	Esp. carinas	INTERMED
**Mesothelioma**	—										
**Gastrointestinal**											
**Esophagus**	*Could be permanent*	Y	Y	Y							
**Gastric / gastric outlet**	*Few alternatives*	Y	N	Y	Y	Y	Y	Y	Y	Needed	HIGH
**Rectum**	*Often unique*	Y	N	Y	Y	Y	Y	Y	N	Probable	HIGH
**Pelvic / Gynecologic**											

Site / Disease	Comments	HDR	LDR	Perm vLDR	BTX	Hypertx	Drain	Chemo	Immunotx	Notes	Enthusiasm
**Opportunities to improve patient care** — *Work in progress, subject to continuous improvement*											
**Cervix**	*Alternatives*	Y	N	Y	Y	Y	Y	N	N		HIGH
**Peritoneal surface**	*Creative planar*										
**Urogenital**											
**Ureter (rare)**	*Luminal*	N	N	Y	Y	N	Y	N	N		INTERMED
**Pancreatic**											
**Pancreatic ducts**	*Luminal*	N	N	Y	Y	Y	Y	Y	N		INTER

Legend

Y / N Modality is applicable (Y) or not currently applicable (N) for the listed site.

? Applicability uncertain; warrants further investigation.

— Section header or category placeholder; no per-modality assessment intended.

[1] Possibly applicable in the future; tentatively classified as no at present.

HORM Hormonal therapy applicable as an adjunct.

Modality abbreviations

HDR High dose rate brachytherapy

LDR Low dose rate brachytherapy

Perm vLDR Permanent / very low dose rate brachytherapy

BTX Brachytherapy device or boost (general)

Hypertx Hyperthermia

Drain Drainage / pressure modulation via device

Chemo Local chemotherapy delivery via device

Immunotx Local immunotherapy delivery via device

HORM Hormonal therapy as an adjunct

Notes

Radioactive isotopes that remain in place over the course of a few days deliver, in effect, a low dose rate (LDR) treatment.

The enthusiasm ratings reflect the authors' subjective clinical assessment of priority for further investigation; HIGH indicates strong unmet need with reasonable device fit; INTERMED / INTER indicates promising but more contingent.

This list is illustrative rather than exhaustive. It is intended to stimulate discussion and collaboration across radiation oncology, surgical, and engineering disciplines.
